# Coping with sport trauma and well-being in athletes

**DOI:** 10.1192/j.eurpsy.2021.1231

**Published:** 2021-08-13

**Authors:** A. Yavorovskaya, S. Leonov, E. Rasskazova

**Affiliations:** 1 Faculty Of Psychology, Lomonosov Moscow State University, Moscow, Russian Federation; 2 Clinical Psychology, Moscow State University, Moscow, Russian Federation

**Keywords:** Coping Strategies, well-being of athletes, sport trauma

## Abstract

**Introduction:**

Sport trauma is a stressful situation demanding not only physical but also psychological rehabilitation (Clement et al, 2015, Ardern et al, 2012) including prevention of mental health symptoms. Revealing coping strategies that are related not only to rehabilitation effectiveness but also to well-being of athletes is important for rehabilitation programs (Crowther et al, 2017, Hamson-Utley, Vazquez, 2008, Johnston, Carroll, 1998).

**Objectives:**

The aim was to reveal coping strategies that are related to better well-being in athletes after trauma after adjusting for trauma perception.

**Methods:**

61 athletes (15-25 years old, 31 males) rehabilitating after sport trauma filled COPE with specific instruction about trauma (Carver et al., 1989), Illness Perception Questionnaire (Moss-Morris et al., 2002) modified for trauma situation, Satisfaction with Life Scale (Diener et al., 1985), Scale of Positive and Negative Experience (Diener et al., 2009).

**Results:**

After adjusting for subjective trauma representation humor related to trauma predicted better satisfaction with life (β=.43, R^2^=43.7%). Active coping with trauma was related to more positive emotions (β=.31, R^2^=9.8%) while emotion venting, substance use and lower instrumental support are related to negative emotions (β=.39, β=.24, β=-.29, respectively, R^2^=30.5%), although these effects eliminate after adjusting for trauma representation.

**Conclusions:**

Humor, active coping and instrumental support as well as control of emotion venting and substance use could be helpful strategies of promotion of better well-being in athletes after sport trauma. Research is supported by the Russian Science Foundation, project No. 19-78-10134.

**Conflict of interest:**

Research is supported by the Russian Science Foundation, project No. 19-78-10134.

